# SNR Wall Effect Alleviation by Generalized Detector Employed in Cognitive Radio Networks

**DOI:** 10.3390/s150716105

**Published:** 2015-07-03

**Authors:** Modar Safir Shbat, Vyacheslav Tuzlukov

**Affiliations:** School of Electronics Engineering, College of IT Engineering, Kyungpook National University, 1370 Sankyuk-dong, Buk-gu, Daegu 702-701, Korea; E-Mail: modboss80@gmail.com

**Keywords:** cognitive radio (CR), spectrum sensing, generalized detector (GD), energy detector (ED), noise uncertainty, sample complexity, antenna array, *SNR* wall

## Abstract

The most commonly used spectrum sensing techniques in cognitive radio (CR) networks, such as the energy detector (ED), matched filter (MF), and others, suffer from the noise uncertainty and signal-to-noise ratio (*SNR*) wall phenomenon. These detectors cannot achieve the required signal detection performance regardless of the sensing time. In this paper, we explore a signal processing scheme, namely, the generalized detector (GD) constructed based on the generalized approach to signal processing (GASP) in noise, in spectrum sensing of CR network based on antenna array with the purpose to alleviate the *SNR* wall problem and improve the signal detection robustness under the low *SNR*. The simulation results confirm our theoretical issues and effectiveness of GD implementation in CR networks based on antenna array.

## 1. Introduction

**Figure 1:**
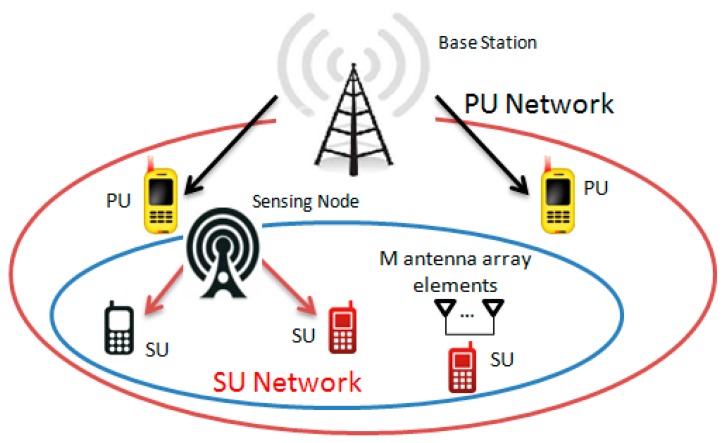
CR systems and SU with *M* antenna array elements.

The main aim of the cognitive radio (CR) network is to improve the spectrum utilization efficiency, by introducing an opportunistic use of unemployed frequency band by the primary user (PU) (see Figure 1). The spectrum sensing is needed to define the frequency holes that could be allocated for the secondary user (SU). The spectrum sensors search continuously an availability of frequency holes and assign them to SU without causing harmful interference to the PU. Fundamental limitations in practice are involved in spectrum sensing process [1,2,3]. The sensitivity to noise power uncertainty, for example, variations in the noise variance as a function of real time, is one of the most common and serious problems among the well-known spectrum sensors such as the energy detector (ED), matched filter (MF), and even the cyclostationary detector under some conditions at the low signal-to-noise ratio (*SNR*) [4]. The impact of noise power uncertainty is quantified by *SNR* wall location, *i.e.*, if the *SNR* value is less than the *SNR* wall, the PU signal detector will fail to achieve the desired performance and maintain a robustness against power noise uncertainty independently of how long the sensing time is [3,4,5]. Both theoretical and experimental analysis confirmed the *SNR* wall phenomenon existence under the noise power uncertainty conditions. This phenomenon negatively effects the receiver operation characteristic (ROC). Other uncertainties also can be considered as *SNR* wall generators, for example, the noise power estimation error, assumptions made under the white and stationary noise, fading process, shadowing, non-ideal filters, non-precise analog-to-digital (A/D) converters, quantization noise, aliasing effect caused by imperfect front-end filters, and interference between the PU and SU. An alternative presentation for the *SNR* wall is given by the number of samples *N* as a function of *SNR*, the probability of false alarm $P_{FA}$ and probability of miss $P_{miss}$, *i.e.*, $N=f(SNR,P_{FA},P_{miss})$. The PU signal detector should minimize the number of samples *N* required to achieve the desired detection performance. The lowest *SNR* satisfying the probability of false alarm $P_{FA}$ and the probability of miss $P_{miss}$ constraints is called the detector sensitivity [3].

In general, the ideal ED does not have the *SNR* wall, but owing to the noise power uncertainty the ED suffers from the *SNR* wall phenomenon making the ED non-robust under the low *SNR* [6,7]. In many published papers, the ED spectrum sensing performance is investigated under the noise uncertainty conditions. Different solutions are presented in the form of the dynamic detection threshold [8], log-normal approximation of the noise uncertainty [9], falling the *SNR* wall using the cross-correlation [10], improving the noise power estimation using the maximum likelihood (ML) estimator [6], *SNR* estimation based on the pseudo bit error rate (BER) for the modified ED [11], and algebraic spike detection method introduced in [12,13]. In fact, the best non-coherent detector is non-robust as the ED under the noise power uncertainty. In the coherent detector case, the *SNR* wall is pushed back only to a limited value and for a large channel coherence time $K_{c}\to \infty$. In the MF case, the *SNR* wall location is proportional to $1/K_{c}$ [3], and in the case of feature detector, the *SNR* wall value is less in comparison with the ED one and scales only as $1/\sqrt{K_{c}}$ with the relevant channel coherence time [3]. An interesting new four-level hypothesis blind detector for spectrum sensing in CR systems is presented in [14]. The proposed detector in [14] reduces the negative effects on the CR system performance, which are forming under the in-phase and quadrature-phase (I/Q) imbalance, based on the orthogonal frequency division multiplexing (OFDM) multiple access scheme, and presents a promising solution for any noise power uncertainties or *SNR* wall problem that could be caused by this I/Q imbalance.

Cooperative spectrum sensing, in the course of which the multiple sensors are involved in cooperative spectrum sensing, demonstrates an effective approach to improve the spectrum sensing performance under several problems such as the noise power uncertainty, multipath fading, shadowing, and receiver uncertainties issues. The cooperative spectrum sensing can also solve the critical energy efficiency issue as shown in [15] where the energy efficient cooperative spectrum sensing is proposed and the optimal scheduling of active time for each spectrum sensor helps to extend the network lifetime. Selective grouping based on the cooperative sensing is discussed in [16] where during the sensing time each sensors group senses different radio channels while sensors in the same group perform the joint detection by the targeted channel. This process assures obtaining the more robust and efficient sensing performance comparing with the individual spectrum sensor case under the noise power uncertainty. 

To mitigate the negative effects of noise power uncertainty at the low *SNR*, an implementation of the generalized detector (GD), which is constructed based on the generalized approach to signal processing (GASP) in noise, for the spectrum sensing in CR networks based on antenna array is proposed. The GD represents a combination of the correlation detector, which is optimal in the Neyman-Pearson (NP) criterion sense when there is *a priori* information about the PU signal parameters, and ED, which is optimal in the NP criterion sense if there is no any *a priori* information about the PU signal parameters that are random [17,18,19]. The GD likelihood ratio test, based on which we can make a decision about the PU signal presence or absence in the process incoming at the SU input, demonstrates a definition of the jointly sufficient statistics of the mean and variance of the likelihood ratio and does not require any information about the PU signal and its parameters [17], ([18], Chapter 3). As was discussed in detail in ([18], Chapter 7, pp. 685–692), the main function of GD energy detector (GD ED) is to detect the PU signal and the main function of the GD correlation detector is to define the detected PU signal parameters and make a decision: the detected signal is the expected PU signal with the required parameters or not.

Note that the conventional correlation detector makes a decision about the PU signal presence or absence in the incoming process based on definition of the mean only of the process incoming at the SU input. The conventional ED defines the decision statistics with respect to PU signal presence or absence at the SU input based on determination of the variance only of the process incoming at the SU input. Definition of the jointly sufficient statistics of the mean and variance based of the incoming process at the SU input allows us to make more accurate decision in favor of the PU signal presence or absence and obtain more information about the PU signal parameters under GD employment in CR networks in comparison with the conventional MF, ED, correlation receiver and so on.

A great difference between the GD ED and conventional ED is a presence of the additional linear system (the additional bandpass filter at the GD input) considered as the secondary data or reference noise source. The PU signal bandwidth is mismatched with the additional linear system bandwidth. The PU signal bandwidth is matched only with another linear system bandwidth at the GD front-end. Thus, the GD has two input linear systems, namely, the preliminary filter (PF) and the additional filter (AF). The last is considered as the reference noise source ([18], Chapter 3), [19]. The GD PF central frequency is detuned relatively to the GD AF central frequency to ensure, firstly, the PU signal passing only through GD PF and, secondly, the independence and uncorrelatedness between the stochastic processes at the GD PF and AF outputs. Thus, it is possible to obtain the PU signal plus noise at the GD PF output in the case of “a yes” PU signal at the GD input and only the noise in the opposite case. Consequently, only the noise is obtained at the GD AF output for both cases of “a yes” and “a no” PU signal at the GD input, in other words, under the hypotheses $H_{1}$ and $H_{0}$. The case when there is the PU signal generated by another source with the frequency content within the limits of the GD AF bandwidth, and considered as the additional interference, is discussed in [20]. The GD employment in wireless communications [21,22], radar sensor systems [20,23], and CR networks for spectrum sensing [24] allows us to improve the signal detection performance of these systems in comparison with implementation of widely used conventional detectors. 

This work differs from the previously published paper [24] by introducing a new advantage of GD employing in CR network systems based on antenna array, which is the *SNR* wall problem alleviation under the noise power uncertainty. Additionally, the GD optimal detection threshold is defined based on the minimal probability of error criterion under the noise power uncertainly at the low *SNR* condition. Intuitive approach to reduce the noise power uncertainty at run time by employing the GD in CR network is to define the noise power at the GD AF output, *i.e.*, the another narrow band closed to the PU signal frequency band, with the purpose to calibrate the noise power in the PU signal frequency band. Even if we believe that the noise power forming at the GD PF and AF outputs is not the same, the noise calibration error can be much lower than the noise power uncertainty itself. The noise power calibration in real time improves the immunity against the *SNR* wall phenomenon [3]. In this paper, we investigate the GD noise power calibration effects on the *SNR* wall problem in coarse spectrum sensing for CR network systems based on antenna array and we define the GD sample complexity under the noise power uncertainty. The complementary receiver operating characteristic (ROC) and sample complexity of the ED, MF, and GD are compared under the same initial conditions for different uncertainty parameters. The real scenario of simulation demonstrates that the GD is able to alleviate the *SNR* wall problem and achieve the low probability of error in comparison with the conventional ED.

The reminder of this paper is organized as follows. Section 2 presents the system model and the GD test statistics. Section 3 delivers the GD signal detection performance under the noise power uncertainty. The real scenario simulation results are discussed in Section 4. The concluding remarks are presented in Section 5. 

## 2. System Model and GD Test Statistics

### 2.1. System Model

The spectrum sensor has an antenna array with the number of elements equal to *M* and each antenna array element receives *N* samples during the sensing time. The spectrum sensing problem can be modeled as the conventional binary hypothesis test:

(1) $$\{\begin{array}{c} H_{0}\Rightarrow z_{i}[k]=w_{i}[k],\;i=1,...,M;\;k=0,...,N-1\\ H_{\text{1}}\Rightarrow z_{i}[k]=h_{i}[k]s[k]+w_{i}[k],\;i=1,...,M;\;k=0,...,N-1 \end{array}$$

where $z_{i}[k]$ is the discrete-time received signal at the spectrum sensor input; $w_{i}[k]$ is the discrete-time circularly symmetric complex Gaussian noise with zero mean and variance $\sigma_{w}^{2},$ *i.e.*, $w_{i}[k]\sim CN(0,\sigma_{w}^{2})$; $h_{i}[k]$ is the discrete-time channel coefficients obeying the circularly symmetric complex Gaussian distribution with zero mean and variance equal to $\sigma_{h}^{2}$, *i.e*., $h_{i}[k]\sim CN(0,\sigma_{h}^{2})$; and s[k] is the discrete-time PU signal, *i.e.*, the signal to be detected. We consider the same initial conditions with respect to s[k] as in [3]. The channel parameters are not varied during the sensing time and the channel coefficients $h_{i}[k]$ are spatially correlated between each other. Throughout this paper, the PU signal s[k], the channel coefficients $h_{i}[k]$, and the noise $w_{i}[k]$ are independent and uncorrelated between each other. The same channel model is widely used in [25,26,27]. In general, the ED does not require channel state information (CSI) for spectrum sensing [28] and the GD shares this property with ED because the ED is a constituent of the GD. It is well known that information about the CSI allows us to obtain better spectrum sensing performance in comparison with unknown CSI case. The knowledge about CSI can be more useful and effective in the cooperative spectrum sensing case. Under the low SNR and noise power uncertainty conditions, we can claim that we have imperfect CSI [29]. When the noise power estimation is applied, we have partial knowledge about the CSI. In this paper, we assume that the coarse spectrum sensing is performed without knowledge about the CSI. 

Owing to its simplicity, the exponential matrix model is widely used to describe the spatial correlation between the adjacent antenna array elements [30]. The components of the M×M antenna array element correlation matrix **C** can be presented in the following form:

(2) $${\text{C}}_{ij}=\{{\text{ρ}}^{i-j}\},\;i\leq j,\;i,j=1,......,M$$

where ρ is the coefficient of spatial correlation between the adjacent antenna array elements (0≤ρ≤1, the real values). Applying the results presented in [30], the coefficient of spatial correlation ρ can be given as

(3) $$\text{ρ}=\exp\{-23{\text{Λ}}^{2}{\left(d/\lambda \right)}^{2}\}$$

where Λ is the angular spread, an important propagation parameter defining a distribution of multipath power of radio waves coming in at the receiver input from a number of azimuthal directions with respect to the horizon; λ is the wavelength; and *d* is the distance between two adjacent antenna array elements (the antenna array element spacing). The correlation matrix of antenna array elements **C** given by Equation (2) is the symmetric Toeplitz matrix [25].

We define the NM×1 signal vector **Z** that collects all the observed signal samples during the sensing time using the following form:

(4) $$\text{Z}={\left[z_{1}[0],...,z_{M}[0],\;...\text{ ,}z_{1}[N-1],...,z_{M}[N-1]\right]}^{\;T}$$

where *T* denotes a transpose. The data distribution in the complex matrix **Z** can be expressed as:

(5) $$\text{Z}\sim \{\begin{array}{c} CN(0,\sigma^{2}\text{I})\;,\;\Rightarrow \;H_{\text{0}}\\ CN(0,E_{s}\sigma_{h}^{2}\text{I}+\sigma^{2}\text{I})\;,\;\Rightarrow \;H_{\text{1}} \end{array}$$

where $E_{s}$ is the average energy of transmitted signal at the spectrum sensor input, and **I** is the MN×MN identity matrix. We consider a situation when the primary signaling scheme is unknown (the PU has a total freedom of choosing the signaling strategy). Thus, the detector should be able to detect a presence of any possible PU signal satisfying the power and bandwidth constraints.

The received signal vector **Z** has a complex Gaussian distribution with the covariance matrices $Cov_{0}$ and $Cov_{1}$ under the hypotheses $H_{0}$ and $H_{1},$ respectively. If $z_{i}[k]=w_{i}[k]$, the received signals $z_{i}[k]$ are independent between each other. Under the hypothesis $H_{1}$, when $z_{i}[k]=h_{i}[k]s[k]+w_{i}[k]$, the received signals are spatially correlated. The covariance matrices $Cov_{0}$ and $Cov_{1}$ can be determined in the following form:

(6) $$\left\{ \begin{array}{l} \;Cov_{0}=E[\text{Z}{\text{Z}}^{H}|H_{0}]=\sigma_{w}^{2}\text{I}\\ \;Cov_{1}=E[\text{Z}{\text{Z}}^{H}|H_{1}]=E_{s}\sigma_{h}^{2}A+\sigma_{w}^{2}\text{I} \end{array}\right.$$

where E[⋅] is the mathematical expectation; *H* denotes the Hermitian conjugate (conjugate transpose); **I** is the MN×MN identity matrix; $E_{s}$ is the PU signal energy at the SU input; and A is the MN×MN matrix defined based on the correlation matrix **C** given by Equation (2) [30]:

(7) $$A={\left[\begin{array}{cccc} C & 0_{M} & ... & 0_{M}\\ 0_{M} & \ddots & \ddots & \vdots \\ \vdots & \ddots & \ddots & 0_{M}\\ 0_{M} & ... & 0_{M} & C \end{array}\right]}_{MN\times MN}$$

where $0_{M}$ is the M×M zero matrix.

### 2.2. GD Statistics

The GD has been constructed based on the (GASP) in noise discussed in detail in [17,18,19]. The GD is considered as a linear combination of the correlation detector, which is optimal in the Neyman-Pearson criterion sense under detection of signals with *a priori* known parameters, and the ED, which is optimal in the Neyman-Pearson criterion sense under detection of signals with *a priori* unknown or random parameters. The main functioning principle of GD is a complete matching between the model signal generated by the local oscillator in GD and the information signal, in particular, the PU signal at the GD input by whole range of parameters. In this case, the noise component of the GD correlation detector caused by interaction between the model signal generated by the local oscillator in GD and the input noise and the random component of the GD ED caused by interaction between the incoming information signal (the PU signal) and input noise are cancelled in the statistical sense. This GD feature allows us to obtain the better detection performance in comparison with other classical receivers or detectors.

The specific feature of GASP is introduction of the additional noise source that does not carry any information about the incoming signal with the purpose to improve a qualitative signal detection performance. This additional noise can be considered as the reference noise without any information about the PU signal [17]. The jointly sufficient statistics of the mean and variance of the likelihood ratio is obtained in the case of GASP implementation, while the classical and modern signal processing theories can deliver only a sufficient statistics of the mean or variance of the likelihood ratio. Thus, the implementation of GASP allows us to obtain more information about the input process or received information signal (the PU signal). Owing to this fact, an implementation of receivers constructed based on the GASP basis allows us to improve the spectrum sensing performance of CR wireless networks in comparison with employment of other conventional receivers at the sensing node.

**Figure 2:**
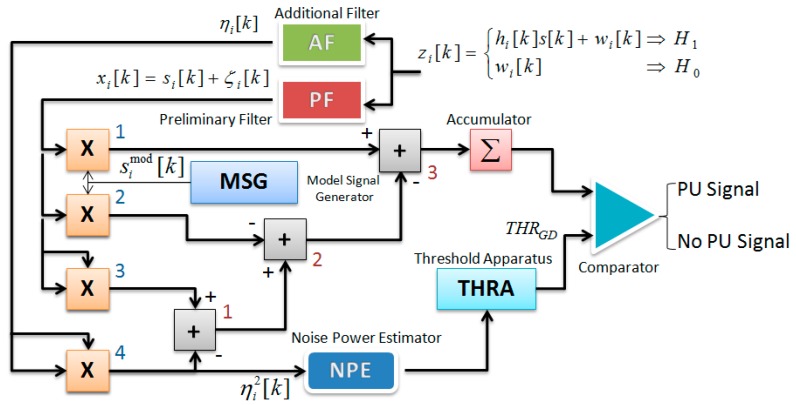
GD flowchart.

The GD flowchart is presented in Figure 2. As we can see from Figure 2, the GD consists of three channels:

• The GD correlation channel—the PF, multipliers 1 and 2, model signal generator MSG;
• The GD ED channel—the PF, AF, multipliers 3 and 4, summator 1;
• The GD compensation channel—the summators 2 and 3 and accumulator Σ.

As follows from Figure 2, under the hypothesis $H_{\text{1}}$ (a “yes” PU signal), the GD correlation channel generates the signal component $s_{i}^{\mathrm{mod}}[k]s_{i}[k]$ caused by interaction between the model signal, the reference signal at the GD model signal generator (MSG) output, and the incoming information signal (the PU signal) and the noise component $2s_{i}^{\mathrm{mod}}[k]\xi_{i}[k]$ caused by interaction between the model signal $s_{i}^{\mathrm{mod}}[k]$ and the noise $\xi_{i}[k]$ (the PF output). Under the hypothesis $H_{\text{1}}$, the GD ED generates the information signal energy $s_{i}^{2}[k]$ and the random component $2s_{i}[k]\xi_{i}[k]$ caused by interaction between the information signal $s_{i}[k]$ and the noise $\xi_{i}[k]$. The main purpose of the GD compensation channel is to cancel in the statistical sense the GD correlation channel noise component $2s_{i}^{\mathrm{mod}}[k]\xi_{i}[k]$ and the GD ED random component $2s_{i}[k]\xi_{i}[k]$ between each other based on the same nature of the noise $\xi_{i}[k]$.

To describe the GD flowchart we consider the discrete-time processes without loss of any generality. Evidently, the cancelation in the statistical sense between the GD correlation channel noise component $2s_{i}^{\mathrm{mod}}[k]\xi_{i}[k]$ and the GD ED random component $2s_{i}[k]\xi_{i}[k]$ is possible only based on the same nature of the noise $\xi_{i}[k]$ satisfying the condition of equality between the signal model $s_{i}^{\mathrm{mod}}[k]$ and incoming PU signal $s_{i}[k]$ over the whole range of parameters. The condition

(8) $$s_{i}^{\mathrm{mod}}[k]=s_{i}[k]$$

is the main GD functioning condition. Naturally, in practice, the signal parameters are random. The complete matching between the model signal $s_{i}^{\mathrm{mod}}[k]$ and the incoming signal $s_{i}[k]$ (the PU signal), especially by amplitude, is a very hard problem in practice and only in the ideal case the complete matching is possible. How the GR sensing performance can be deteriorated under mismatching between the model signal $s_{i}^{\mathrm{mod}}[k]$ and the incoming (PU) signal $s_{i}[k]$ is discussed in this paper. 

Under the hypothesis $H_{\text{0}}$, *i.e*., a “no” information signal (the PU signal), satisfying the GD main functioning condition given by (8), we obtain only the background noise $\eta_{i}^{2}[k]-\xi_{i}^{2}[k]$ at the GD output. The GD PF bandwidth is matched with the bandwidth of the information signal (the PU signal) *s_i_*[*k*]. The threshold apparatus (THRA) device defines the GD threshold.

**Figure 3:**
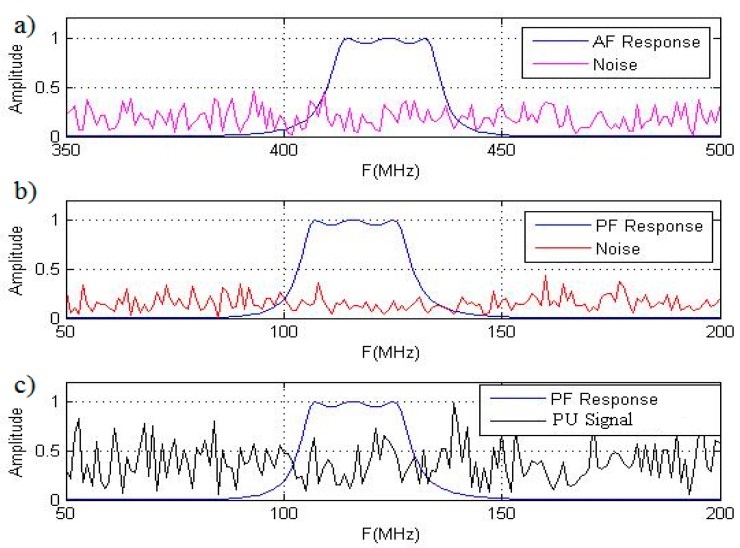
Signals at the GD PF and AF outputs: (**a**) GD AF response and noise; (**b**) GD PF response and noise; (**c**) GD PF response and PU signal.

The GD PF and AF can be considered as the linear systems, for example, as the bandpass filters, with the impulse responses $h_{PF}[m]$ and $h_{AF}[m]$, respectively. For simplicity of analysis, we assume that these filters have the same amplitude-frequency characteristics or impulse responses by shape. Moreover, the GD AF central frequency is detuned with respect to the GD PF central frequency on such a value that the information signal (the PU signal) cannot pass through the GD AF. Thus, the PU signal and noise can appear at the GD PF output and the only noise is appeared at the GD AF output (see Figure 3). If a value of detuning between the GD AF and PF central frequencies is more than 4 or 5 $\text{Δ}f_{s}$, where $\text{Δ}f_{s}$ is the PU signal bandwidth, the processes at the GD AF and PF outputs can be considered as the uncorrelated and independent processes and, in practice, under this condition, the coefficient of correlation between GD PF and AF output processes is not more than 0.05 that was confirmed experimentally [31,32].

In the present paper, we consider the spectrum sensing problem of a single radio channel where the GD AF bandwidth is always idle and cannot be used by the SU because it is out of the useful spectrum of the PU network. There is a need to note that in a general case, the GD AF portion of the spectrum may be occupied by the PU signals from other networks and can be not absolutely unoccupied. In this case, the PU signals from other networks can be considered as interferences or interfering signals. Investigation and study of GD under this case is discussed in [20].

The processes at the GD AF and PF outputs present the input stochastic samples from two independent frequency-time regions. If the noise w[k] at the GD PF and AF inputs is Gaussian, the noise at the GD PF and AF outputs is Gaussian, too, because the GD PF and AF are the linear systems, and we believe that these linear systems do not change the statistical parameters of the input process. We use this assumption for simplicity of theoretical analysis. Thus, the GD AF can be considered as a reference noise source with *a priori* knowledge a “no” signal (the reference noise sample). Detailed discussion of the GD AF and PF can be found in [18,19]. The noise at the GD PF and AF outputs can be presented in the following form:

(9) $$\{\begin{array}{c} w_{PF}[k]=\sum_{i=1}^{M}\xi_{i}[k]=\sum_{m=-\infty }^{\infty }h_{PF}[m]w_{i}[k-m]\\ w_{AF}\text{[}k\text{]}=\sum_{i=1}^{M}\eta_{i}[k]=\sum_{m=-\infty }^{\infty }h_{AF}\text{[}m\text{]}w_{i}[k-m]\; \end{array}$$

Under the hypothesis $H_{1}\text{,}$ the signal at the GD PF output can be defined as $x_{i}[k]=s_{i}[k]+\xi_{i}[k]$ (see Figure 2), where $\xi_{i}[k]$ is the observed noise at the GD PF output and

(10) $$s_{i}[k]=h_{i}[k]\times s[k]$$

Under the hypothesis $H_{0}\text{,}$ and for all *i* and *k*, the process $x_{i}[k]=\xi_{i}[k]$ at the GD PF output is subjected to the complex Gaussian distribution and can be considered as the independent and identically distributed (i.i.d.) process. The process at the GD AF output is the reference noise $\eta_{i}[k]$ with the same statistical parameters as the noise $\xi_{i}[k]$ in the ideal case. We make this assumption for simplicity. In practice, the statistical parameters of the noise $\xi_{i}[k]$ and $\eta_{i}[k]$ are different, as a rule. We consider this case below. The decision statistics at the GD output presented in [17,18] is extended to the case of antenna array employment when an adoption of multiple antennas and antenna arrays is effective to mitigate the negative attenuation and fading effects [20,24]. The GD decision statistics can be presented in the following form:

(11) $$T_{GD}(X)=\sum_{k=0}^{N-1}\sum_{i=1}^{M}2x_{i}[k]s_{i}^{\mathrm{mod}}[k]-\sum_{k=0}^{N-1}\sum_{i=1}^{M}x_{i}^{2}[k]+\sum_{k=0}^{N-1}\sum_{i=1}^{M}\eta_{i}^{2}[k]\underset{{<}_{H_{0}}}{{>}^{H_{1}}}THR_{GD}$$

where

(12) $$X={\left[x_{1}[0],\ldots ,x_{M}[0],\;\ldots \text{ ,}x_{1}[N-1],\ldots ,x_{M}[N-1]\right]}^{T}$$

is the stochastic process vector at the GD PF output and $THR_{GD}$ is the GD detection threshold. We can rewrite Equation (11) in the vector form:

(13) $$T_{GD}(X)=2S^{\mathrm{mod}}X-X^{2}+\eta^{2}\underset{{<}_{\;H_{0}}}{{>}^{H_{1}}}THR_{GD}$$

where

(14) X=[x(0),...,x(N−1)]

is the M×1 vector of the random process at the GD PF output with elements defined as

(15) $$x[k]={\left[x_{1}[k],\ldots ,x_{M}[k]\right]}^{T}$$

(16) $$S^{\mathrm{mod}}=[s^{\mathrm{mod}}(0),\ldots ,s^{\mathrm{mod}}(N-1)]$$

is the M×1 vector of the process at the MSG output with the elements defined as

(17) $$s^{\mathrm{mod}}[k]={\left[s_{1}^{\mathrm{mod}}[k],\ldots ,s_{M}^{\mathrm{mod}}[k]\right]}^{T}$$

(18) η=[η(0),…,η(N−1)]

is the M×1 vector of the random process at the AF output with the elements defined as

(19) $$\eta [k]={\left[\eta_{1}[k],\ldots ,\eta_{M}[k]\right]}^{T}$$

and $THR_{GD}$ is the GD detection threshold. According to GASP and GD structure shown in Figure 2 and the main GD functioning condition (8), the GD test statistics takes the following form under the hypotheses $H_{1}$ and $H_{0}$, respectively:

(20) $$T_{GD}(X)=\left\{ \begin{array}{l} \sum_{k=0}^{N-1}\sum_{i=1}^{M}s_{i}^{2}[k]+\sum_{k=0}^{N-1}\sum_{i=1}^{M}\eta_{i}^{2}[k]-\sum_{k=0}^{N-1}\sum_{i=1}^{M}\xi_{i}^{2}[k]\;\Rightarrow H_{1}\\ \sum_{k=0}^{N-1}\sum_{i=1}^{M}\eta_{i}^{2}[k]-\sum_{k=0}^{N-1}\sum_{i=1}^{M}\xi_{i}^{2}[k]\;\Rightarrow H_{0}\; \end{array}\right.$$

The term $\sum_{k=0}^{N-1}\sum_{i=1}^{M}s_{i}^{2}[k]$ is the average energy of received signal and the term $\sum_{k=0}^{N-1}\sum_{i=1}^{M}\eta_{i}^{2}[k]-$ $\sum_{k=0}^{N-1}\sum_{i=1}^{M}\xi_{i}^{2}[k]$ presents the background noise at the GD output that is a difference between the noise power at the GD PF and AF outputs. It is important to mention that the GD main functioning condition is the equality between parameters of the model signal $s_{i}^{\mathrm{mod}}[k]$ and the PU signal $s_{i}[k]$ (see Equation (8)) over all range of parameters and, in particular, by amplitude. How we can satisfy this condition in practice is discussed in detail in [17] and ([18], Chapter 6, pp. 611–621 and Chapter 7, pp. 631–695) when there is no *a priori* information about the signal $s_{i}[k]$. Additionally, a practical implementation of the GD decision statistics requires an estimation of the noise variance $\sigma_{w}^{2}$ using the reference noise $\eta_{i}[k]$ at the GD AF output.

The mean $m_{H_{0}}^{GD}$ and variance $Var_{H_{0}}^{GD}$ of the test decision statistics $T_{GD}(X)$ under the hypothesis $H_{0}$ are given in the following form ([19], Chapter 3):

(21) $$\left\{ \begin{array}{l} \;m_{H_{0}}^{GD}=E\text{[}T_{GD}(X)|H_{0}]=0\\ \;Var_{H_{0}}^{GD}=Var\text{[}T_{GD}(X)|H_{0}]=4NM\sigma^{4} \end{array}\right.$$

The above-mentioned discussion is correct for the case when the noise variances at the GD AF and GD PF outputs are the same, *i.e*., $\sigma_{\xi }^{2}=\sigma_{\eta }^{2}=\sigma^{2}$. For the case that is very close to practice if the GD AF and PF are the bandpass filters with deviation in parameters, *i.e*., $\sigma_{\xi }^{2}\neq \sigma_{\eta }^{2}$, we can assume $\sigma_{\xi }^{2}=\sigma^{2}$ and $\sigma_{\eta }^{2}=$ $\beta \sigma_{\xi }^{2}=\beta \sigma^{2}$, where β is the noise coefficient (or factor) of proportionality. For this case, (21) takes the following form:

(22) $$\left\{ \begin{array}{l} \;m_{H_{0}}^{GD}=E\text{[}T_{GD}(X)|H_{0}]=0\\ \;Var_{H_{0}}^{GD}=Var\text{[}T_{GD}(X)|H_{0}]=2NM\sigma^{4}(1+\beta^{2}) \end{array}\right.$$

We use representation Equation (22) in the following discussion, for example, in Section 3.

### 2.3. Moment Generation Function of the GD Partial Test Statistics $T_{GD}(X_{k})$

To define the mean $m_{H_{1}}^{GD}$ and variance $Var_{H_{1}}^{GD}$ of the test statistics $T_{GD}(X)$ under the hypothesis $H_{1}$, the moment generation function (MGF) of the GD partial test statistics $T_{GD}(X_{k})$ given by

(23) $$T_{GD}(X_{k})=\sum_{i=1}^{M}s_{i}^{2}[k]+\sum_{i=1}^{M}\eta_{i}^{2}[k]-\sum_{i=1}^{M}\xi_{i}^{2}[k]$$

is required. The MGF of the GD partial test statistics $T_{GD}(X_{k})$ is presented as:

$$M_{T_{GD}(X_{k})}(l)=\prod_{i=1}^{M}\frac{1}{1-E_{s}\sigma_{h}^{2}\alpha_{i}l}\prod_{i=1}^{M}M_{z_{1_{i}}}(l)\prod_{i=1}^{M}M_{z_{2_{i}}}(-l)=\prod_{i=1}^{M}\frac{1}{\sqrt{\left(1-2\sigma^{2}l)(1+2\sigma^{2}l\right)}}\times \frac{1}{1-E_{s}\sigma_{h}^{2}\alpha_{i}l}$$

(24) $$=\frac{1}{\sqrt{{\left(1-4\sigma^{4}l\right)}^{M}}}\prod_{i=1}^{M}\frac{1}{1-E_{s}\sigma_{h}^{2}\alpha_{i}l}$$

Derivation of Equation (24) in detail is given in Appendix A. 

Based on Equation (24), the mean $m_{H_{1}}^{GD}$ and the variance $Var_{H_{1}}^{GD}$ of the test statistics $T_{GD}(X)$ under the hypothesis $H_{1}$ take the following form, respectively:

(25) $$m_{H_{1}}^{GD}=E\text{[}T_{GD}(X_{k})|H_{1}]=NME_{s}\sigma_{h}^{2}$$

(26) $$Var_{H_{1}}^{GD}=Var\text{[}T_{GD}(X_{k})|H_{1}]=N\left[\sum_{i=1}^{M}E_{s}^{2}\sigma_{h}^{4}\alpha_{i}^{2}+4M\sigma^{4}\;\right]$$

For the case $\sigma_{\xi }^{2}\neq \sigma_{\eta }^{2}$, Equations (25) and (26) take the following form

(27) $$m_{H_{1}}^{GD}=E\text{[}T_{GD}(X_{k})|H_{1}]=NME_{s}\sigma_{h}^{2}$$

(28) $$Var_{H_{1}}^{GD}=Var\text{[}T_{GD}(X_{k})|H_{1}]=N\left[\sum_{i=1}^{M}E_{s}^{2}\sigma_{h}^{4}\alpha_{i}^{2}+2M\sigma^{4}(1+\beta^{2}\text{) }\right]$$

## 3. GD Spectrum Sensing and Sample Complexity

The spectrum sensor should minimize the number of samples *N*, *i.e.*, the sample complexity, required to distinguish the hypotheses $H_{0}$ and $H_{1}$ with high accuracy under definite constraints applied to the standard and desired probability of false alarm $P_{FA}$ and probability of miss $P_{miss}$. For example, according to the IEEE 802.22 standards, the constraints are $P_{FA}\leq 0.1$ and $P_{miss}\leq 0.1$ [33]. 

### 3.1. The Case $s_{i}^{m}[k]=s_{i}[k]$

For the considered case, Equations (24)–(28) are valid. We assume that the received PU signal and noise at the GD input are independent. Thus, as N→∞ the central limit theorem is valid and can be applied. The probability density function (pdf) of the GD test statistics $T_{GD}(X)$ can be approximated by the normal Gaussian distribution. In this case, the probability of false alarm $P_{FA}^{GD}$ and the probability of miss $P_{miss}^{GD}$ can be expressed in the following form [19]:

(29) $$\left\{ \begin{array}{l} \;P_{FA}^{GD}=Q\left(\frac{THR_{GD}-m_{H_{0}}^{GD}}{\sqrt{Var_{H_{0}}^{GD}}}\right)\;=Q\left(\frac{THR_{GD}}{2\sigma^{2}\sqrt{NM}}\right)\text{ , }\sigma_{\xi }^{\text{2}}=\sigma_{\eta }^{\text{2}}=\sigma^{2}\\ \;P_{FA}^{GD}=Q\left(\frac{THR_{GD}-m_{H_{0}}^{GD}}{\sqrt{Var_{H_{0}}^{GD}}}\right)\;=Q\left(\frac{THR_{GD}}{\sqrt{2NM\sigma^{4}(1+\beta^{2})\;}}\right)\text{ , }\sigma_{\xi }^{\text{2}}\neq \sigma_{\eta }^{\text{2}} \end{array}\right.$$

(30) $$\left\{ \begin{array}{l} \;P_{miss}^{GD}=1-Q\;\left(\frac{THR_{GD}-m_{H_{1}}^{GD}}{\sqrt{Var_{H_{1}}^{GD}}}\right)\;=1-Q\left(\frac{THR_{GD}-NME_{s}\sigma_{h}^{2}}{\sqrt{NM[E_{s}^{2}\sigma_{h}^{4}+4\sigma^{4}]}}\right)\text{ , }\sigma_{\xi }^{\text{2}}=\sigma_{\eta }^{\text{2}}={\text{σ}}^{2}\;\\ \;P_{miss}^{GD}=1-Q\;\left(\frac{THR_{GD}-m_{H_{1}}^{GD}}{\sqrt{Var_{H_{1}}^{GD}}}\right)\;=1-Q\left(\frac{THR_{GD}-NME_{s}\sigma_{h}^{2}}{\sqrt{NM[E_{s}^{2}\sigma_{h}^{4}+2\sigma^{4}(1+\beta^{2})]}}\right)\text{ , }\sigma_{\xi }^{\text{2}}\neq \sigma_{\eta }^{2} \end{array}\right.$$

where

(31) $$Q(x)=\frac{1}{\sqrt{2\pi }}\int_{x}^{\infty }\exp(-0.5t^{2})dt$$

is the Gaussian Q-function.

**Figure 4:**
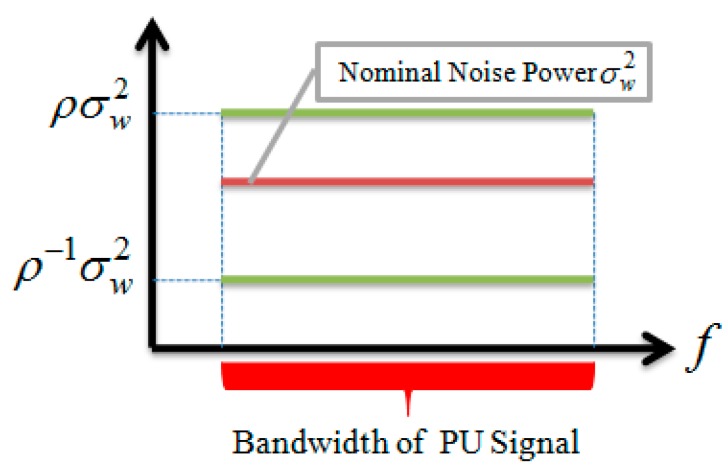
Power noise uncertainty model.

In the noise power uncertainty case, the noise power or variance at the GD PF and AF outputs can be determined only within the limits of a definite range [3] (see Figure 4)

(32) $$\sigma^{2}\in [{\text{ρ}}^{-1}\sigma_{w}^{2},\text{ρ}\sigma_{w}^{2}]$$

*i.e.*, the actual noise power is bounded by the lower and upper bounds, where $\sigma_{w}^{2}$ is the nominal noise power or variance at the GD input

(33) $$\text{ρ}=10^{0.1\text{ε}}$$

is the uncertainty parameter; ε is the parameter used to define the amount of non-probabilistic uncertainty in the noise power. 

In the case of noise power uncertainty, Equations (29) and (30) can be written in the following form:

(34) $$\left\{ \begin{array}{l} \;P_{FA}^{GD}=\underset{\sigma^{2}\in [{\text{ρ}}^{-1}\sigma_{w}^{2},\text{ρ}\sigma_{w}^{2}]}{\max}Q\left(\frac{THR_{GD}}{\sqrt{4NM\sigma^{4}}}\right)\;=Q\left(\frac{THR_{GD}}{2\text{ρ}\sigma_{w}^{2}\sqrt{NM}}\right)\text{ , }\sigma_{\xi }^{\text{2}}=\sigma_{\eta }^{2}=\sigma^{2}\\ \;P_{FA}^{GD}=\underset{\sigma^{2}\in [{\text{ρ}}^{-1}\sigma_{w}^{2},\text{ρ}\sigma_{w}^{2}]}{\max}Q\left(\frac{THR_{GD}}{\sqrt{2(\sigma_{\xi }^{4}+\sigma_{\eta }^{4})NM}}\right)\;=Q\left(\frac{THR_{GD}}{\text{ρ}\sigma_{w}^{2}\sqrt{2NM(1+\beta^{2})}}\right)\text{ , }\sigma_{\xi }^{\text{2}}\neq \sigma_{\eta }^{2} \end{array}\right.\;$$

(35) $$\left\{ \begin{array}{l} \;P_{miss}^{GD}=1-\underset{\sigma^{2}\in [{\text{ρ}}^{-1}\sigma_{w}^{2},\text{ρ}\sigma_{w}^{2}]}{\min}Q\left(\frac{THR_{GD}-NME_{s}\sigma_{h}^{2}}{\sqrt{NM[E_{s}^{2}\sigma_{h}^{4}+4\sigma^{4}]}}\right)\;\\ \;=1-Q\left(\frac{THR_{GD}-NME_{s}\sigma_{h}^{2}}{{\text{ρ}}^{-1}\sigma_{w}^{2}\sqrt{NM(SNR^{2}+4)}}\right)\text{ , }\sigma_{\xi }^{\text{2}}=\sigma_{\eta }^{2}=\sigma^{2}\\ \;P_{miss}^{GD}=1-\underset{\sigma^{2}\in [{\text{ρ}}^{-1}\sigma_{w}^{2},\text{ρ}\sigma_{w}^{2}]}{\min}Q\left(\frac{THR_{GD}-NME_{s}\sigma_{h}^{2}}{\sqrt{NM[E_{s}^{2}\sigma_{h}^{4}+2(\sigma_{\xi }^{4}+\sigma_{\eta }^{4})]}}\right)\;\\ \;=1-Q\left\{\frac{THR_{GD}-NME_{s}\sigma_{h}^{2}}{{\text{ρ}}^{-1}\sigma_{w}^{2}\sqrt{NM[SNR^{2}+2(1+\beta^{2})]}}\right\}\text{ , }\sigma_{\xi }^{\text{2}}\neq \sigma_{\eta }^{2}\; \end{array}\right.$$

where

(36) $$SNR=\frac{E_{s}\sigma_{h}^{2}}{\sigma_{w}^{2}}$$

is the *SNR* at the GD input. Based on Equations (34) and (35), the threshold $THR_{GD}$ can be defined as

(37) $$\left\{ \begin{array}{l} \;THR_{GD}=2\text{ρ}\sigma_{w}^{2}\sqrt{NM}Q^{-1}(P_{FA}^{GD})\text{ ; }\sigma_{\xi }^{\text{2}}=\sigma_{\eta }^{2}=\sigma^{2}\\ \;THR_{GD}=\text{ρ}\sigma_{w}^{2}\sqrt{2NM(1+\beta^{2})}\;Q^{-1}(P_{FA}^{GD})\text{ ; }\sigma_{\xi }^{\text{2}}\neq \sigma_{\eta }^{2} \end{array}\right.$$

As SNR<<1, substituting Equation (37) in Equation (35), the GD sample complexity can be defined as

(38) $$N_{GD}=\left\{ \begin{array}{l} \;\frac{4{\left[\text{ρ}Q^{-1}(P_{FA}^{GD})-{\text{ρ}}^{-1}Q^{-1}(1-P_{\text{miss}}^{GD})\right]}^{2}}{M{\left(SNR\right)}^{2}}\text{ ; }\sigma_{\xi }^{\text{2}}=\sigma_{\eta }^{2}=\sigma^{2}\\ \;\frac{2(1+\beta^{2}){\left[\text{ρ}Q^{-1}(P_{FA}^{GD})-{\text{ρ}}^{-1}Q^{-1}(1-P_{\text{miss}}^{GD})\right]}^{2}}{M{\left(SNR\right)}^{2}}\text{ ; }\sigma_{\xi }^{\text{2}}\neq \sigma_{\eta }^{2} \end{array}\right.$$

Here we assume that $\sigma_{\xi }^{2}\in [{\text{ρ}}^{-1}\sigma_{w}^{2},\text{ρ}\sigma_{w}^{2}]$ and $\sigma_{\eta }^{2}\in [{\text{ρ}}^{-1}\sigma_{w}^{2},\text{ρ}\sigma_{w}^{2}]$. As follows from Equation (38), the sample complexity $N_{GD}$ is inversely proportional to the squared *SNR*.

We can notice that there is no additional term involving the *SNR* in the denominator of Equation (38) which leads to the noise power uncertainty calibration, *i.e.*, the *SNR* wall alleviation. This is caused by the complete compensation in the ideal case between the noise component $2s_{i}^{\mathrm{mod}}[k]\xi_{i}[k]$ of the GD correlation channel and the random component $2s_{i}[k]\xi_{i}[k]$ of the GD ED channel and also because the mean $m_{H_{0}}^{GD}$ of the test statistics $T_{GD}(X)$ is equal to zero under the hypothesis $H_{0}$ [17]. This result confirms an effectiveness of the GD test statistics under the use of reference noise forming at the GD AF output for *SNR* wall alleviation.

The relation between the probability of miss $P_{miss}^{GD}$ and probability of false alarm $P_{FA}^{GD}$ is given by

(39) $$\left\{ \begin{array}{l} \;P_{miss}^{GD}=1-Q\left[{\text{ρ}}^{2}Q^{-1}(P_{FA}^{GD})-\frac{\text{ρ}}{2}\sqrt{NM}\;SNR\right]\text{ ; }\sigma_{\xi }^{\text{2}}=\sigma_{\eta }^{2}=\sigma^{2}\\ \;P_{miss}^{GD}=1-Q\left[2(1+\beta^{2}){\text{ρ}}^{2}Q^{-1}(P_{FA}^{GD})-\frac{\text{ρ}}{\sqrt{2}}SNR\sqrt{NM(1+\beta^{2})}\;\right]\text{ ; }\sigma_{\xi }^{\text{2}}\neq \sigma_{\eta }^{2} \end{array}\right.$$

Following the above-mentioned procedure we can obtain the sample complexity for ED, which can be determined in the following form:

(40) $$N_{ED}=\frac{{\left[\text{ρ}Q^{-1}(P_{FA}^{ED})-{\text{ρ}}^{-1}Q^{-1}(1-P_{miss}^{ED})\right]}^{2}}{M{\left[SNR-(\text{ρ}-{\text{ρ}}^{-1})\right]}^{2}}$$

As follows from (40), we can define the ED *SNR* wall in the following form [3]

(41) $$SNR_{wall}^{ED}=\frac{{\text{ρ}}^{2}-1}{\text{ρ}}$$

The relation between the probability of miss $P_{miss}^{ED}$ and probability of false alarm $P_{FA}^{ED}$ can be defined as

(42) $$P_{miss}^{ED}=1-Q\{{\text{ρ}}^{2}Q^{-1}(P_{FA}^{ED})-\text{ρ}\sqrt{NM}[SNR-(\text{ρ}-{\text{ρ}}^{-1})]\}$$

In the MF case, the effective *SNR* is provided by the coherent processing gain. Thus, the MF sample complexity is given by [3]

(43) $$N_{MF}=\frac{2K_{c}{\left[Q^{-1}(P_{FA}^{MF})-Q^{-1}(1-P_{miss}^{MF})\right]}^{2}}{M{\left[\text{θ }K_{c}SNR-(\text{ρ}-{\text{ρ}}^{-1})\right]}^{2}}$$

where $K_{c}$ is the coherence time of the radio channel, *i.e.*, the time interval, within the limits of which the channel impulse response is not varied; θ is a fraction of the total power that is allocated to the known pilot tone. This concept covers many practical wireless communication systems employing the pilot tones and training known sequences for synchronization and timing acquisition. The MF *SNR* wall can be presented in the following form [3]:

(44) $$SNR_{wall}^{MF}=\frac{1}{\text{θ }K_{c}}\frac{{\text{ρ}}^{2}-1}{\text{ρ}}\;$$

The ED has the better sample complexity performance at the high *SNR* in comparison with the MF because the ED uses the total average PU signal power for detection while the MF uses only a fraction of the total PU signal power. In the case of MF possessing the pilot tone detection scheme, the *SNR* wall phenomenon is a consequence of time-selectivity of the channel fading process and the signal power is increased with the factor $K_{c}$ owing to increasing the coherent processing gain. This is the reason why we see that the MF is sensitive to the channel coherence time $K_{c}$. Thus, the effective *SNR* of the coherently combined signal according to [1,2] is given by

(45) $$SNR_{eff}^{MF}=\text{θ }K_{c}SNR$$

### 3.2. The Case $s_{i}^{m}[k]\neq s_{i}[k]$

In practice, the problem to satisfy an equality between the model signal $s_{i}^{\mathrm{mod}}[k]$ and incoming PU signal $s_{i}[k]$ amplitudes is a very difficult problem. For simplicity, we consider a case when a relation between the amplitudes of the model signal $s_{i}^{\mathrm{mod}}[k]$ and incoming PU signal $s_{i}[k]$ can be presented in the following form:

(46) $$s_{i}^{\mathrm{mod}}[k]=\text{μ}s_{i}[k]$$

where μ is the amplitude coefficient of proportionality. Under the condition given by Equation (46), the MGF of the GD partial decision statistics

(47) $$T_{GD}(X_{k})=\sum_{i=1}^{M}s_{i}^{2}[k](2\text{μ}-1)+\sum_{i=1}^{M}2s_{i}[k]\zeta_{i}[k](\text{μ}-1)+\sum_{i=1}^{M}\eta_{i}^{2}[k]-\sum_{i=1}^{M}\xi_{i}^{2}[k]$$

takes the following form:

(48) $$M_{T_{GD}(X_{k})}(l)=\frac{1}{\sqrt{{\left(1-4\sigma^{4}l\right)}^{M}}}\times \prod_{i=1}^{M}\frac{1}{1-E_{s}\sigma_{h}^{2}(2\text{μ}-1)\alpha_{i}l}\times \prod_{i=1}^{M}\frac{1}{1-2(\text{μ}-1)\alpha_{i}l\sqrt{E_{s}\sigma_{h}^{2}\sigma^{2}}}$$

Based on Equation (48), the mean $m_{H_{1}}^{GD}$ and the variance $Var_{H_{1}}^{GD}$ of the test statistics $T_{GD}(X)$ under the hypothesis $H_{1}$ are defined using the following form:

(49) $$\begin{array}{l} \left\{ \begin{array}{l} \;m_{H_{1}}^{GD}=\text{E}\left[T_{GD}(X)|H_{1}\right]=NM(2\text{μ}-1)E_{s}\sigma_{h}^{2}\text{ ;}\\ \;Var_{H_{1}}^{GD}=Var\left[T_{GD}(X)|H_{1}\right]=NM\{{\left[(2\text{μ}-1)E_{s}\sigma_{h}^{2}\right]}^{2}+4{\left(\text{μ}-1\right)}^{2}E_{s}\sigma_{h}^{2}\sigma^{2}+4\sigma^{4}\text{\} ; } \end{array}\right.\sigma_{\xi }^{2}=\sigma_{\eta }^{2}=\sigma^{2}\\ \left\{ \begin{array}{l} \;m_{H_{1}}^{GD}=\text{E}\left[T_{GD}(X)|H_{1}\right]=NM(2\text{μ}-1)E_{s}\sigma_{h}^{2}\text{ ;}\\ \;Var_{H_{1}}^{GD}=Var\left[T_{GD}(X)|H_{1}\right]\\ \;=NM\{{\left[(2\text{μ}-1)E_{s}\sigma_{h}^{2}\right]}^{2}+2{\left(\text{μ}-1\right)}^{2}E_{s}\sigma_{h}^{2}(\sigma_{\xi }^{2}+\sigma_{\eta }^{2})+2(\sigma_{\xi }^{4}+\sigma_{\eta }^{4})\}\text{ ; } \end{array}\right.\;\sigma_{\xi }^{2}\neq \sigma_{\eta }^{2} \end{array}$$

In the case of noise power uncertainty and under the condition given by Equation (46), Equation (49) allows us to define the probability of false alarm $P_{FA}^{GD}$ and the probability of miss $P_{miss}^{GD}$ for GD using the following form:

(50) $$\left\{ \begin{array}{l} \;P_{FA}^{GD}=Q\left(\frac{THR_{GD}}{2\text{ρ}\sigma_{w}^{2}\sqrt{NM}}\right)\text{ ; }\sigma_{\xi }^{\text{2}}=\sigma_{\eta }^{2}=\sigma^{2}\\ \;P_{FA}^{GD}=Q\left(\frac{THR_{GD}}{\text{ρ}\sigma_{w}^{2}\sqrt{2NM(1+\beta^{2})}}\right)\text{ ; }\sigma_{\xi }^{\text{2}}\neq \sigma_{\eta }^{2} \end{array}\right.$$

(51) $$\left\{ \begin{array}{l} \;P_{miss}^{GD}=1-Q\left(\frac{THR_{GD}-NM(2\text{μ}-1)E_{s}\sigma_{h}^{2}}{2{\text{ρ}}^{-1}\sigma_{w}^{2}\sqrt{NM[{\left(\text{μ}-1\right)}^{2}SNR^{2}+1]}}\right)\text{ ; }\sigma_{\xi }^{\text{2}}=\sigma_{\eta }^{2}=\sigma^{2}\\ \;P_{miss}^{GD}=1-Q\left(\frac{THR_{GD}-NM(2\text{μ}-1)E_{s}\sigma_{h}^{2}}{{\text{ρ}}^{-1}\sigma_{w}^{2}\sqrt{NM[{\left(\text{μ}-1\right)}^{2}SNR^{2}+2(1+\beta^{2})]}}\right)\text{ ; }\sigma_{\xi }^{\text{2}}\neq \sigma_{\eta }^{2} \end{array}\right.$$

Under delivering (51) we ignore the term ${\left(2\text{μ}-1\right)}^{2}SNR^{2}$ since CR networks operate at very low *SNR* values, *i.e.*, SNR<<1. Defining the threshold $THR_{GD}$ in terms of the probability of false alarm $P_{FA}^{GD}$ based on (50) and substituting it in (51), we obtain

(52) $$\left\{ \begin{array}{l} \;P_{miss}^{GD}=1-Q\left(\frac{2\text{ρ}Q^{-1}(P_{FA}^{GD})-\sqrt{NM}(2\text{μ}-1)SNR}{2{\text{ρ}}^{-1}\sqrt{{\left(\text{μ}-1\right)}^{2}SNR^{2}+1}}\right)\text{ ; }\sigma_{\xi }^{\text{2}}=\sigma_{\eta }^{2}=\sigma^{2}\\ \;P_{miss}^{GD}=1-Q\left(\frac{\sqrt{2(1+\beta^{2})}\text{ ρ}Q^{-1}(P_{FA}^{GD})-\sqrt{NM}(2\text{μ}-1)SNR}{{\text{ρ}}^{-1}\sqrt{\left[{\left(\text{μ}-1\right)}^{2}SNR^{2}+2(1+\beta^{2})\right]}}\right)\text{ ; }\sigma_{\xi }^{\text{2}}\neq \sigma_{\eta }^{2} \end{array}\right.$$

At the low *SNR* values, we can apply the following approximation ${\left(\text{μ}-1\right)}^{2}SNR^{2}+1\approx 1$ and determine the GD sample complexity using the following form:

(53) $$\left\{ \begin{array}{l} \;N_{GD}=\frac{4{\left[\text{ρ}Q^{-1}(P_{FA}^{GD})-{\text{ρ}}^{-1}Q^{-1}(1-P_{miss}^{GD})\right]}^{2}}{M{\left(2\text{μ}-1\right)}^{2}{\left(SNR\right)}^{2}}\text{ ; }\sigma_{\xi }^{\text{2}}=\sigma_{\eta }^{2}=\sigma^{2}\\ \;N_{GD}=\frac{2(1+\beta^{2}){\left[\text{ρ}Q^{-1}(P_{FA}^{GD})-\;{\text{ρ}}^{\text{-1}}Q^{-1}(1-P_{miss}^{GD})\right]}^{2}}{M{\left(2\text{μ}-1\right)}^{2}{\left(SNR\right)}^{2}}\text{ ; }\sigma_{\xi }^{\text{2}}\neq \sigma_{\eta }^{2} \end{array}\right.$$

At μ=1 we obtain the sample complexity $N_{GD}$ given by Equation (38).

### 3.3. The GD Optimal Threshold

As a matter of fact, the ED and GD ignore the PU signal characteristics and rely only on the PU signal energy. Thus, the ED and GD optimal threshold should be proportional to the nominal noise power at the SU input. In practice, the noise power is unknown and should be estimated by the GD noise power estimator (NPE in Figure 2). As a result, both the ED and GD detection thresholds can be defined based on the total error rate minimization [34,35,36]. In the case of the additive white Gaussian noise (AWGN) channel, the GD optimal threshold can be defined using the minimal probability of error in the following form:

(54) $$THR_{GD}^{op}=\arg\underset{THR_{GD}}{\min}P_{er}^{GD}(THR_{GD})$$

where $P_{er}^{GD}$ is the probability of error given by

(55) $$P_{er}^{GD}=P(H_{0})P_{FA}^{GD}+P(H_{1})P_{miss}^{GD}$$

where $P(H_{0})$ and $P(H_{1})=1-P(H_{0})$ are the *a priori* probabilities of the PU signal absence or presence, respectively. For simplicity of analysis, we assume that these *a priori* probabilities are known and equal to $P(H_{0})=P(H_{1})=0.5$. The optimal GD threshold can be expressed as (see Appendix B)

(56) $$\left\{ \begin{array}{l} \;THR_{GD}^{op}=\frac{4NM\sigma_{w}^{2}}{1+{\text{ρ}}^{-2}}\text{ ; }\sigma_{\xi }^{\text{2}}=\sigma_{\eta }^{2}=\sigma^{2}\\ \;THR_{GD}^{op}=\frac{4NM\sigma_{w}^{2}(1+\beta^{2})}{2(1+{\text{ρ}}^{-2})}\text{ ; }\sigma_{\xi }^{\text{2}}\neq \sigma_{\eta }^{2} \end{array}\right.$$

As we can see from (56), in the ideal case, *i.e*., when there is no noise power uncertainty ρ=1 and β=1 or $\sigma_{\xi }^{\text{2}}=\sigma_{\eta }^{2}=\sigma^{2}$, the optimal detection threshold is determined as

(57) $$THR_{GD}^{op}=2NM\sigma_{w}^{2}$$

In practice, in the GD case, there is no need to define or know *a priori* the value of ρ since the noise power is estimated in the real time using the NPE (see Figure 2). We consider the optimal threshold under the noise power uncertainty for the theoretical analysis presented in this paper.

Since the estimated noise power is differed from the real noise power, the noise power uncertainty is an unavoidable problem in practice [2,3,37]. As discussed in [6,38], in the ED case, the *SNR* wall phenomenon is caused by insufficient refinement of the noise power estimation while the observation time is increased and the noise power estimation approach can avoid the *SNR* wall problem if the noise power estimate is consistent within the limits of the observation interval. Finally, we cannot rely on the noise power estimation to solve the *SNR* wall problem. The results presented in [6] are applicable for ED under the use of the noise power estimation and can be applied to GD implementation in CR networks.

## 4. Simulation and Discussion

The sample complexity and existence of the *SNR* wall for the ED and MF and non-existence of the *SNR* wall for the GD are verified by the real scenario simulation that is performed using MATLAB in accordance with the parameters presented in the IEEE 802.22 standards, *i.e.*, the standard for wireless regional area network WRAN using white spaces in the TV broadcast bands such as the digital video broadcasting-terrestrial DVB-T. The simulation parameters are presented in Table 1. 

**Table 1:** Main simulation parameters.

Parameter	Value
Number of antenna array elements	M=2; 6
Signal-to-noise ratio	SNR=−40÷0 [dB]
Probability of false alarm	$P_{FA}=0.1$
Probability of miss	$P_{miss}=0.1$
Non-probabilistic uncertainty parameter	ε=1; 0.1 ; 0.001 [dB]
Channel parameter	$\sigma_{h}^{2}=1$
Channel coherence time	$K_{c}=100;\;1000$
Fraction of the total power	θ=0.1

**Figure 5:**
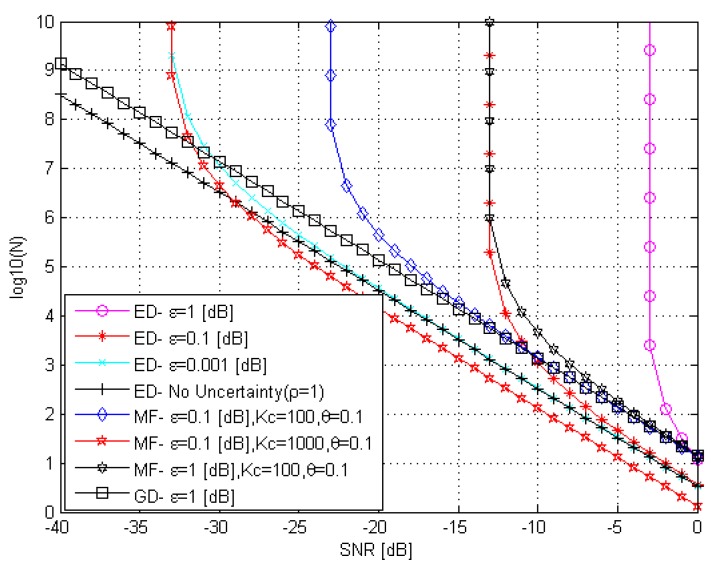
The sample complexity curves of ED, MF, and GD under the noise power uncertainty.

As we can see from Figure 5, in the ideal case, *i.e.*, the complete compensation of the noise component of the GD correlation channel $2s_{i}^{\mathrm{mod}}[k]\zeta_{i}[k]$ and the random component of the GD ED channel $2s_{i}[k]\xi_{i}[k]$, the GD presents the best sample complexity performance in comparison with the ED and MF under conditions of the noise power uncertainty. The GD overcomes a negative impact of the noise power uncertainty. Thus, the GD can detect the PU signal at any arbitrary low *SNR* increasing the number *N* of samples. In other words, there is no *SNR* wall. In the case of ED, when there is no noise power uncertainty, *i.e*., ρ=1, there is no *SNR* wall and the PU signal can be detected at any low *SNR* by increasing the sensing time or the number *N* of samples. If there is the noise power uncertainty, there is the *SNR* wall for the ED and its location depends on the value of ε and, consequently, the uncertainty parameter ρ. For example, at ε=1 dB, the $SNR_{wall}^{ED}=-3$ dB, and at ε=0.1 dB, the $SNR_{wall}^{ED}=-13$ dB. Thus, the sample complexity tends to approach infinity as the *SNR* decreases tending to approach the *SNR* wall:

(58) $$\underset{SNR^{ED}\to SNR_{wall}^{ED}}{\lim}f(SNR^{ED},P_{FA},P_{miss},\text{ρ})\;\to \infty$$

Small values of ε, the least uncertainty case, are preferred because, in this case, there is a decreasing in *SNR* wall.

The MF has the better sample complexity performance at the low *SNR* in comparison with the ED. For example, at ε=0.1 dB, $SNR_{wall}^{ED}=-13\text{ dB}$ and $SNR_{wall}^{MF}=-23\text{ dB}$. The MF sample complexity depends on the channel coherence time $K_{c}$. As we can see, at the same value of ε, $SNR_{wall}^{MF}$ is decreased if the channel coherence time $K_{c}$ is increased, *i.e.*, $SNR_{wall}^{MF}=-23\text{ dB}$ at $K_{c}=100$ and $SNR_{wall}^{MF}=-33\text{ dB}$ at $K_{c}=1000$.

**Figure 6:**
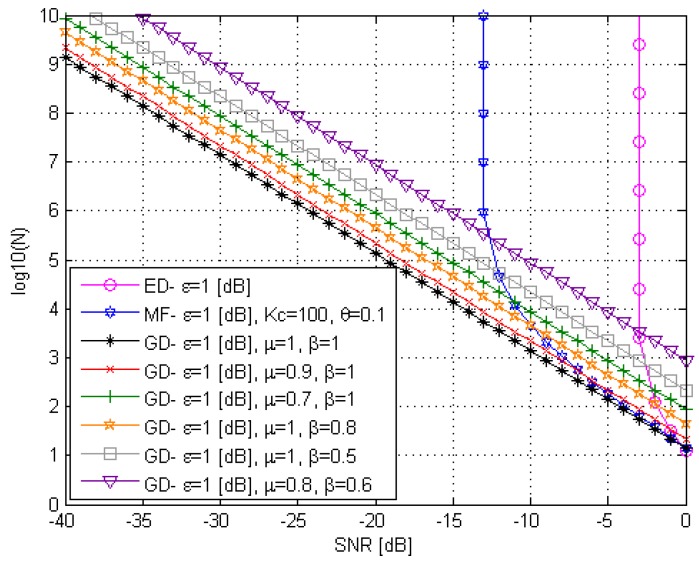
The GD sample complexity at the condition $s_{i}^{\mathrm{mod}}[k]\neq s_{i}[k]$.

The GD sample complexity under the non-ideal condition, the case in practice, $s_{i}^{\mathrm{mod}}[k]\neq s_{i}[k]$ and $\sigma_{\xi }^{\text{2}}\neq \sigma_{\eta }^{2}$, is presented in Figure 6 at ε=1 dB, M=2 and several values of μ and β. As we can see from Figure 6, the best GD sample complexity efficiency is obtained at μ=1 or $s_{i}^{\mathrm{mod}}[k]=s_{i}[k]$ and β=1 or $\sigma_{\xi }^{\text{2}}$ $=\sigma_{\eta }^{2}$. Additionally, we can see that there is no *SNR* wall in the GD case, but the GD sample complexity efficiency decreases at μ≠1 and β≠1. In this case, more samples are needed at the same *SNR* value to achieve the required probability of false alarm.

**Figure 7:**
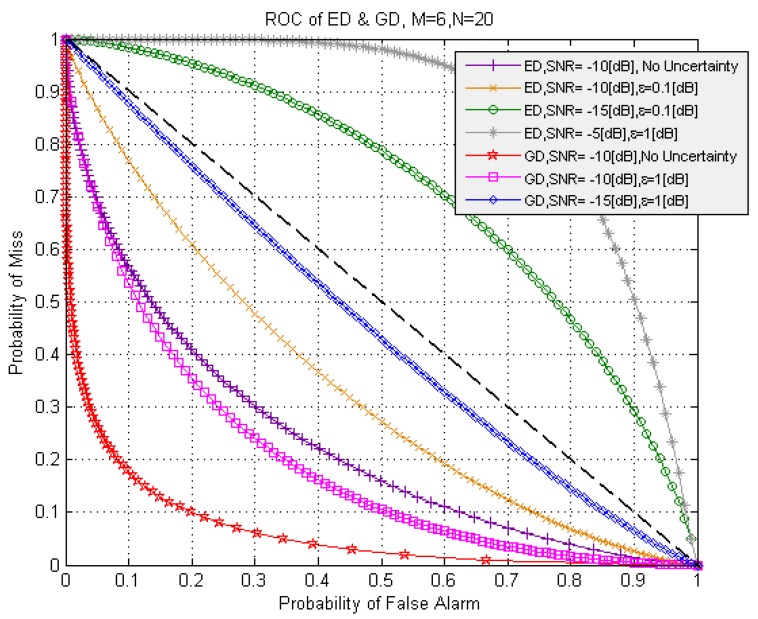
The complementary ROC curves for ED and GD.

The complementary receiver operating characteristic (ROC) curves, which are widely used in practice, for example in [39,40,41], for the ED and GD are presented in Figure 7 with and without the noise power uncertainty at M=6 and N=20. In a general, for both detectors the noise power uncertainty leads to the complementary ROC curves shifting away from the (0,0) origin. As shown in Figure 7, the GD demonstrates the better sensing performance in comparison with the ED and the sensing performance degradation rate of GD is less under the noise power uncertainty conditions. In the GD case, under the low *SNR* or if the *SNR* is above the $SNR_{wall}^{ED}$, the sensing performance degradation caused by the noise power uncertainty can be compensated by increasing in the number of samples *N*. However, if the *SNR* is below the $SNR_{wall}^{ED}$, the ED complementary ROC curve is over the dotted straight line corresponding to the random coin-tossing detector case in Figure 7. This situation is observed at ε=0.1 dB, SNR=−15 dB if SNR=−13 dB (Figure 5) and ε=1 dB, SNR=−5 dB when $SNR_{wall}^{ED}=-3\text{ dB}$ (Figure 6).

**Figure 8:**
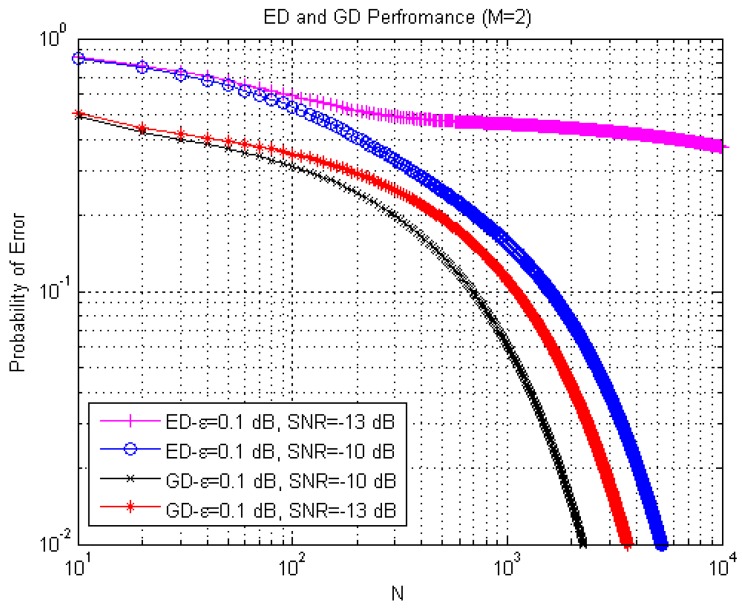
Spectrum sensing performance for the ED and GD in terms of the probability of error $P_{er}$ as a function of the sample number.

In Figure 8, a comparison between the ED and GD performance in terms of the probability of error $P_{er}$ as a function of the sample number *N*, the analogous performance is discussed in [36], is shown at ε=0.1 dB, SNR=−10 dB, and SNR=−13 dB. The GD demonstrates the better sensing performance in comparison with the ED one. For example, at $N=10^{2}$ the probability of error $P_{er}$ is equal to 0.3126 in the GD case and 0.5346 in the ED case. At SNR=−13 dB that corresponds to the $SNR_{wall}^{ED}$ when ε=0.1 dB, we can see that the probability of error $P_{er}$ in the ED case fails to be robust and is distinctly differed owing to the *SNR* wall phenomenon. In this case, an increasing in the number of samples *N* is not effective to improve the probability of error $P_{er}$ performance for ED. At the same time, the GD has the same normal behavior meaning that the probability of error $P_{er}$ performance for GD is improved with increasing in the number of samples *N*.

**Figure 9:**
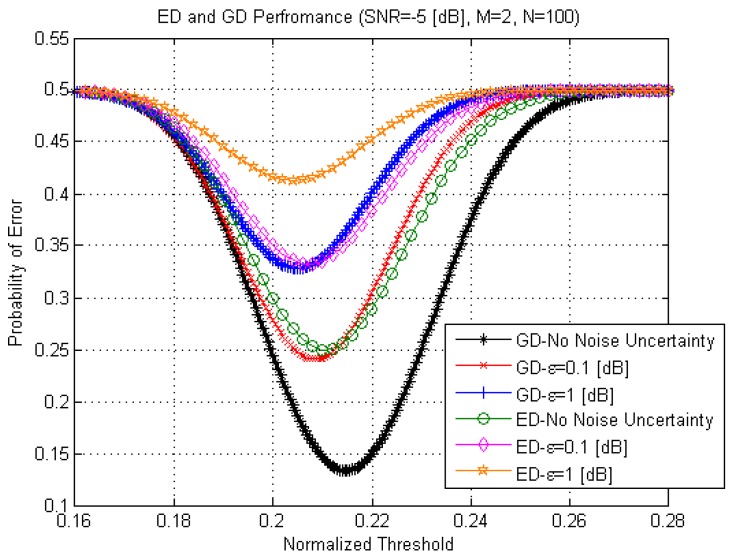
The probabilities of error $P_{er}^{GD}$ and $P_{er}^{ED}$ as a function of the normalized threshold, $\sigma_{\xi }^{\text{2}}=\sigma_{\eta }^{2}=\sigma^{2}$.

Comparison between the probability of error $P_{er}$ for the ED and GD as a function of the normalized optimal detection threshold, where NM is the normalization factor, is presented in Figure 9. The probability of error $P_{er}$ is evaluated for both detectors in two cases: there is the noise power uncertainty and there is no noise power uncertainty at the SNR=−5 dB, M=2, N=100, ε=0.1  and 1 dB. As shown in Figure 9, the GD can achieve the lower probability of error $P_{er}$ in comparison with the ED for both cases. For example, if there is no noise power uncertainty the minimal probability of error $P_{er}$ is equal to 0.13 in the GD case and 0.25 in the ED case. If there is the noise power uncertainty with ε=0.1 dB, the lowest probability of error $P_{er}$ for the GD is equal to 0.24 and 0.33 for the ED. In a general case, the noise power uncertainty affects negatively on the ED and GD probability of error $P_{er}$. Thus, we can make the following conclusion: increasing in the noise power uncertainty leads to increasing in the probability of error $P_{er}$.

**Figure 10:**
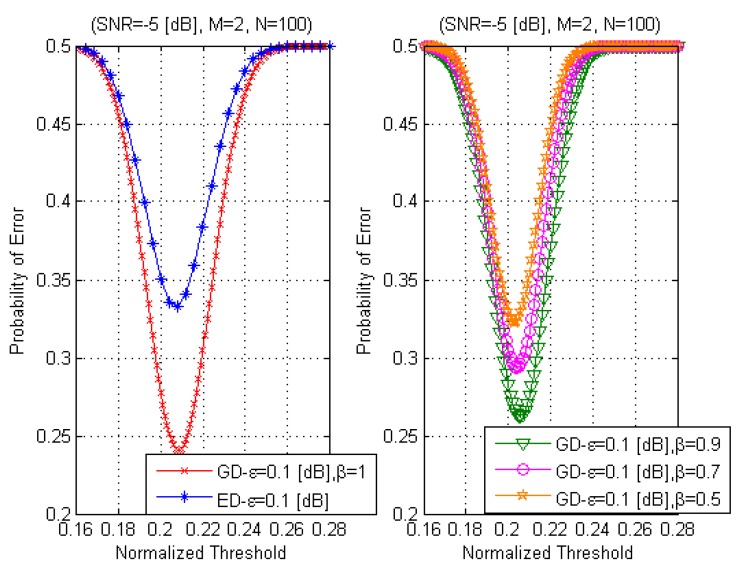
The probabilities of error $P_{er}^{GD}$ and $P_{er}^{ED}$ as a function of the normalized threshold, $\sigma_{\xi }^{\text{2}}\neq \sigma_{\eta }^{2}$.

Figure 10 demonstrates the effect of inequality between the noise variances $\sigma_{\xi }^{\text{2}}\neq \sigma_{\eta }^{2}\;$ at the GD PF and AF outputs on the probability of error $P_{er}$ as a function of the normalized detection threshold given by Equation (56) at SNR=−5 dB, M=2, N=100, ε=0.1 dB. We can notice that the β value effects GD performance. For example, at β=0.9 the probability of error $P_{er}^{GD}$ is approximately equal to 0.26 and at β=0.5 the probability of error $P_{er}^{GD}$ is equal to 0.32. 

As follows form the theoretical analysis and simulation results, the GD implementation allows us to improve the spectrum sensing accuracy that is defined by the probability of false alarm $P_{FA}$ and the probability of detection $P_{D}$. Additionally, the GD allows us to alleviate the *SNR* wall problem by calibrating the noise power uncertainly increasing the number of samples. Thus, the GD employment allows us to improve the signal detection and signal processing performance. The main GD can be applicable in many practical systems, such as the adaptive and spectrum efficient communication systems, CR network systems, and carrier sense multiple access based on wireless networks. In terms of complexity, the GD implementation can be more complicated in comparison with some conventional detectors, for example, the ED. The complexity of GD implementation in practice is caused by the following problems: (1) the inequality between the noise power or variances at the GD PF and AF outputs (discussed in this paper); (2) the problem of matching by parameters between the model signal and the incoming PU signal parameters, for example, by the amplitude or energy (discussed in this paper); (3) the interfering signals within the frequency content of the GD AF, *i.e.*, the GD AF bandwidth (discussed in [20]).

## 5. Conclusions

The actual spectrum sensing performance of the well-known detectors employed in CR networks based on antenna array, such as the ED and MF deviates from the theoretical results owing to the noise power uncertainty and *SNR* wall phenomenon. This phenomenon has a negative impact on the spectrum sensing performance and on the receiver operation characteristic (ROC) when increasing in the sensing time has no any compensating effects. In this paper, we demonstrate that under implementation of the GD in CR networks based on antenna array there is no $SNR_{wall}^{GD}$ in the case of noise power uncertainty that is confirmed by the real scenario simulation. The GD can calibrate the noise power uncertainty problem by the compensation channel (see Figure 2) using the reference noise forming at the GD AF output. The GD is able to detect the PU signal at any low *SNR* value with increasing in the number of samples that is still not the ideal solution under fast spectrum sensing. Thus, the GD implementation in CR networks based on antenna array allows us to reduce some negative effects caused by the noise power uncertainty and improve the PU signal detection performance and robustness. The probability of error $P_{er}$ as a function of the normalized optimal detection threshold is evaluated for the ED and GD both under presence and absence of the noise power uncertainty. The GD demonstrates the better probability of error $P_{er}$ performance in comparison with the ED in both cases. Finally, as is demonstrated by the simulation results, with an increase in the noise power uncertainty, the probability of error $P_{er}$ increases as well. 

## Symbols and Variables Summary

**Symbol or Variable**	**Description**	**Symbol or Variable**	**Description**
ε	Non-probabilistic uncertainty parameter	$m_{H_{0}}^{GD}$	Mean of the GD decision statistics under the hypothesis $H_{0}$
ρ	Coefficient of spatial correlation	$Var_{H_{0}}^{GD}$	Variance of the GD decision statistics under the hypothesis $H_{0}$
θ	Fraction of the total power	$m_{H_{1}}^{GD}$	Mean of the GD decision statistics under the hypothesis $H_{1}$
Λ	Angular spread	$Var_{H_{1}}^{GD}$	Variance of the GD decision statistics under the hypothesis $H_{1}$
λ	Wavelength	$THR_{GD}$	GD detection threshold
β	Noise factor	$THR_{GD}^{op}$	GD optimal detection threshold
μ	Amplitude factor	$P_{FA}$	Probability of false alarm
υ	Chi-square distribution degree of freedom	$P_{miss}$	Probability of miss
$\alpha_{i}$	Eigenvalue of the *i-*th spatial channel of the correlation matrix	$P_{FA}^{GD}$	GD probability of false alarm
$\sigma_{h}^{2}$	Channel parameter	$P_{miss}^{GD}$	GD probability of miss
$\sigma_{w}^{2}$	Noise variance	$P_{FA}^{ED}$	ED probability of false alarm
$\sigma_{\xi }^{2}$	Noise variance at GD PF output	$P_{miss}^{ED}$	ED probability of miss
$\sigma_{\eta }^{2}$	Noise variance at GD AF output	$P_{FA}^{MF}$	MF probability of false alarm
$\text{Δ}f_{s}$	PU signal bandwidth	$P_{miss}^{MF}$	MF probability of miss
$0_{M}$	M×M zero matrix	$P_{er}$	Probability of error
d	Antenna array element spacing	$P_{er}^{GD}$	GD probability of error
C	Antenna array element correlation matrix	$P(H_{0})$	*a priori* probability of the PU signal absence
$Cov_{0}$	Covariance matrix under the hypothesis $H_{0}$	$P(H_{1})$	*a priori* probability of the PU signal presence
$Cov_{1}$	Covariance matrix under the hypothesis $H_{1}$	$SNR_{wall}^{ED}$	ED SNR wall
$E_{s}$	Average energy of transmitted signal	$SNR_{wall}^{MF}$	MF SNR wall
$H_{0}$	Hypothesis of signal absence	$SNR_{eff}^{MF}$	MF effective SNR
$H_{1}$	Hypothesis of signal presence	$w_{i}[k]$	Discrete-time circularly symmetric complex Gaussian noise
$h_{i}[k]$	Discrete-time channel coefficients	X	Stochastic process vector at the PF output
$h_{PF}[m]$	GD PF impulse response	$x_{i}[k]$	Discrete-time signal at PF output
$h_{AF}[m]$	GD AF impulse response	$z_{i}[k]$	Discrete-time received signal
I	Identity matrix	$\xi_{i}[k]$	Noise at PF output
$K_{c}$	Channel coherence time	$\eta_{i}[k]$	Noise at AF output
M	Number of antenna array elements	Z	Signal vector
N	Number of samples (sample complexity)	η	Vector of the random process at the AF output
$N_{GD}$	GD sample complexity	$S^{\mathrm{mod}}$	Vector of the process at the MSG output
$N_{ED}$	ED sample complexity	SNR	Signal-to-noise ratio
$N_{MF}$	MF sample complexity	$T_{GD}(X)$	GD decision statistics
s[k]	PU Signal	$s_{i}^{\mathrm{mod}}[k]$	Model signal

## Appendix A

We say that any random variable *x* has a chi-square distribution with υ degree of freedom if its probability density function (pdf) is presented as

(A1) $$p(x)=cx^{0.5\text{υ}-1}\exp(-0.5x)$$

where *c* is a constant given by [42]

(A2) $$c=\frac{1}{2^{0.5\text{υ}}\text{Γ}(0.5\text{υ})}$$

Γ(⋅) is the gamma function. The MGF form for the chi-square distributed random variable *x* is determined as

(A3) $$M_{x}(l)=E\text{[}\exp(lx)]=\int_{-\infty }^{\infty }\exp(lx)p(x)dx=c\int_{0}^{\infty }\exp(lx)x^{0.5\text{υ}-1}\exp(-0.5x)\;dx$$

At υ=1, the constant *c* can be presented in the following form:

(A4) $$c=\frac{1}{2^{0.5}\text{Γ}(0.5\text{υ})}\;=\frac{\text{1}}{\sqrt{\text{2}\pi }}$$

Assume that $z_{1_{i}}[k]=\xi_{i}^{2}[k]$ and $z_{2_{i}}[k]=\eta_{i}^{2}[k]$. The pdfs for the random variables $z_{1_{i}}$ and $z_{2_{i}}$ are defined by the chi-square $\chi^{2}$ distribution law with one degree of freedom [18]:

(A5) $$p(z_{1_{i}})=\frac{1}{\sqrt{2\pi \;z_{1_{i}}}\;\sigma }\exp\left\{-\frac{z_{1_{i}}}{2\sigma^{2}}\right\}\;,\;z_{1_{i}}>0$$

(A6) $$p(z_{2_{i}})=\frac{1}{\sqrt{2\pi \;z_{2_{i}}}\;\sigma }\exp\left\{-\frac{z_{2_{i}}}{2\sigma^{2}}\right\}\;,\;z_{2_{i}}>0$$

Define a new random variable $z_{i}=z_{1_{i}}-z_{2_{i}}$. The MGF of the random variable $z_{i}$ is given using the following formula:

$$M_{z_{i}}(l)=E\text{[}\exp(lz_{i})]=E\text{\{}\exp[l(z_{1_{i}}-z_{2_{i}})]\}=E\text{[}\exp(lz_{1_{i}})\exp(-lz_{2_{i}})]$$

(A7) $$=E\text{[}\exp(lz_{1_{i}})]E\text{[}\exp(-lz_{2_{i}})]=M_{z_{1_{i}}}(l)M_{z_{2_{i}}}(-l)\;$$

The MGF of the random variable $z_{1_{i}}$ can be defined as

(A8) $$M_{z_{1_{i}}}(l)=\frac{\text{1}}{\sqrt{\text{2}\pi }\;\sigma }\int_{0}^{\infty }\frac{\exp(lz_{1_{i}})}{\sqrt{z_{1_{i}}}}\exp\left\{-\frac{z_{1_{i}}}{2\sigma^{2}}\right\}dz_{1_{i}}=\frac{\text{1}}{\sqrt{\text{2}\pi }\;\sigma }\int_{0}^{\infty }\frac{1}{\sqrt{z_{1_{i}}}}\exp\left\{-\left[\frac{1}{2\sigma^{2}}-l\right]z_{1_{i}}\right\}dz_{1_{i}}$$

Introducing the variable $g_{i}=\frac{z_{1_{i}}}{2\sigma^{2}}-lz_{1_{i}}$, we can write:

(A9) $$M_{z_{1_{i}}}(l)=\frac{2\sigma^{2}}{\sqrt{\text{2}\pi }\sigma }\times \int_{0}^{\infty }\sqrt{\frac{1-2\sigma^{2}l}{2\sigma^{2}}}\frac{1}{\sqrt{g_{i}}}\exp(-g_{i})\frac{1}{1-2\sigma^{2}l}dg_{i}=\sqrt{\frac{1}{\pi (1-2\sigma^{2}l)}}\int_{0}^{\infty }\frac{e^{-g_{i}}}{\sqrt{g_{i}}}dg_{i}$$

Based on definition of the gamma function [42]

(A10) $$\text{Γ}(x)=\int_{0}^{\infty }l^{x-1}\exp(-l)dl$$

we obtain that

(A11) $$\int_{0}^{\infty }\frac{e^{-g_{i}}}{\sqrt{g_{i}}}dg_{i}=\text{Γ}(0.5)=\sqrt{\pi }$$

The final MGF of the random variable $z_{1_{i}}$ is defined as

(A12) $$M_{z_{1_{i}}}(l)=\frac{1}{\sqrt{\;1-2\sigma^{2}l}}$$

The mean and the variance of the random variables $z_{1_{i}}$ can be determined in the following form:

(A13) $$E[z_{1_{i}}]={\frac{\partial M_{z_{1_{i}}}(l)}{\partial l}|}_{l=0}=\sigma^{2}\;$$

(A14) $$Var[z_{1_{i}}]=E[z_{1i}^{2}]-E{\left[z_{1_{i}}\right]}^{2}={\frac{\partial^{2}M_{z_{1_{i}}}(l)}{\partial^{2}l}|}_{l=0}-E{\left[z_{1_{i}}\right]}^{2}=3\sigma^{4}-\sigma^{4}=2\sigma^{4}$$

By the analogous way, we can find that the MGF of the random variable $z_{2_{i}}$ takes the following form:

(A15) $$M_{z_{2_{i}}}(-l)=\frac{1}{\sqrt{\;1+2\sigma^{2}l}}$$

Since ${\left\{s_{i}[k]\right\}}_{i=1}^{M}$ are spatially correlated for *i-*th antenna array elements and according to [43,44], the MGF of $\sum_{i=1}^{M}s_{i}^{2}[k]$ is defined as

(A16) $$M_{\sum_{i=1}^{M}s_{i}^{2}[k]}(l)=\prod_{i=1}^{M}(1-E_{s}\sigma_{h}^{2}\alpha_{i}l{)}^{-1}$$

where $\alpha_{i}$ is the eigenvalue of the *i-*th spatial channel of the correlation matrix **C** given by Equation (2). Based on Equations (A12), (A15) and (A16) the MGF of the GD partial decision statistics $T_{GR}(X_{k})$ is determined in the following form:

$$M_{T_{GD}(X_{k})}(l)=\prod_{i=1}^{M}\frac{1}{1-E_{s}\sigma_{h}^{2}\alpha_{i}l}\prod_{i=1}^{M}M_{z_{1_{i}}}(l)\prod_{i=1}^{M}M_{z_{2_{i}}}(-l)=\prod_{i=1}^{M}\frac{1}{\sqrt{\left(1-2\sigma^{2}l)(1+2\sigma^{2}l\right)}}\times \frac{1}{1-E_{s}\sigma_{h}^{2}\alpha_{i}l}$$

(A17) $$=\frac{1}{\sqrt{{\left(1-4\sigma^{4}l\right)}^{M}}}\prod_{i=1}^{M}\frac{1}{1-E_{s}\sigma_{h}^{2}\alpha_{i}l}$$

## Appendix B

Using the following representation for the *Q*-function [45]

(B1) $$Q(x)=0.5erfc(x/\sqrt{2})$$

and taking into consideration (50), and (51), (54) can be written in the following form:

(B2) $$\left\{ \begin{array}{l} \;THR_{GD}^{op}\\ =\arg\underset{THR_{GD}}{\min}\frac{\text{1}}{\text{2}}\left[1-\frac{1}{2}\text{erfc}\left(\frac{THR_{GD}-NM(2\text{μ}-1)E_{s}\sigma_{h}^{2}}{{\text{ρ}}^{-1}\sigma_{w}^{2}\sqrt{NM(SNR^{2}+4)}}\right)+\frac{1}{2}\text{erfc}\left(\frac{THR_{GD}}{2\text{ρ}\sigma_{w}^{2}\sqrt{NM}}\right)\right]\;\\ \;\sigma_{\xi }^{\text{2}}=\sigma_{\eta }^{2}=\sigma_{w}^{2}\text{ ;}\\ \;THR_{GD}^{op}\\ =\arg\underset{THR_{GD}}{\min}\frac{\text{1}}{\text{2}}\left[1-\frac{1}{2}\text{erfc}\left(\frac{THR_{GD}-NM(2\text{μ}-1)E_{s}\sigma_{h}^{2}}{{\text{ρ}}^{-1}\sigma_{w}^{2}\sqrt{NM[SNR^{2}+2(1+\beta^{2})]}}\right)+\frac{1}{2}\text{erfc}\left(\frac{THR_{GD}}{\text{ρ}\sigma_{w}^{2}\sqrt{2NM(1+\beta^{2})}}\right)\right]\;\\ \;\sigma_{\xi }^{\text{2}}\neq \sigma_{\eta }^{2}\text{ ;} \end{array}\right.$$

where

(B3) $$erfc(x)=\frac{2}{\sqrt{\pi }}\int_{x}^{\infty }e^{-t^{2}}dt\;$$

is the complementary error function.

We can notice form Equation (B2) that the probability of error $P_{er}^{GD}(THR_{GD})\;$ as a function of $THR_{GD}$ is twice differentiable for this threshold and based on discussion in [46] we can conclude that $\frac{\partial^{2}P_{er}^{GD}(THR_{GD})}{\partial {\left(THR_{GD}\right)}^{2}}$ >0. Thus, the probability of error $P_{er}^{GD}(THR_{GD})\;$ is a convex function and has a global minimum if the following equality is satisfied $THR_{GD}=THR_{GD}^{op}$. To be more accurate, the probability of error $P_{er}^{GD}(THR_{GD})\;$ is a quasi-convex function since it can have flat regions as we will see later. This implies that we have only one value of the threshold $THR_{GD}$ minimizing the probability of error $P_{er}^{GD}$. This value is the optimal threshold $THR_{GD}^{op}$. We can determine the optimal threshold $THR_{GD}^{op}$ solving the following equation with respect to $THR_{GD}$ using the procedure discussed in [35]:

(B4) $$\frac{1}{\partial (THR_{GD})}\partial [P_{er}^{GD}(THR_{GD})]=0$$

Taking into consideration the following equation [45]

(B5) $$\frac{\partial }{\partial x}erfc\left(\frac{x-a}{b}\right)=-\frac{2}{b\sqrt{\pi }}\exp\left[-\frac{{\left(x-a\right)}^{2}}{b^{2}}\right]$$

where the values *a* and *b* are the arbitrary constants, we can represent Equation (B4) using the following form:

(B6) $$\left\{ \begin{array}{l} \;\frac{1}{\partial (THR_{GD})}\partial [P_{er}^{GD}(THR_{GD})]\\ =\frac{1}{2{\text{ρ}}^{-1}\sigma_{w}^{2}\sqrt{\pi NM(SNR^{2}+4)}}\exp\left[-\frac{{\left[THR_{GD}-NM(2\text{μ}-1)E_{s}\sigma_{h}^{2}\right]}^{2}}{{\text{ρ}}^{-2}NM\sigma_{w}^{4}(SNR^{2}+4)}\right]\\ -\frac{1}{4\text{ρ}\sigma_{w}^{2}\sqrt{\pi NM}}\exp\left[-\frac{THR_{GD}^{2}}{4{\text{ρ}}^{2}NM\sigma_{w}^{4}}\right]\;=\text{0 ; }\sigma_{\xi }^{\text{2}}=\sigma_{\eta }^{2}=\sigma^{2}\;\\ \;\frac{1}{\partial (THR_{GD})}\partial [P_{er}^{GD}(THR_{GD})]\\ =\frac{1}{{\text{ρ}}^{-1}\sigma_{w}^{2}\sqrt{\pi NM[SNR^{2}+2(1+\beta^{2})]}}\exp\left[-\frac{{\left[THR_{GD}-NM(2\text{μ}-1)E_{s}\sigma_{h}^{2}\right]}^{2}}{{\text{ρ}}^{-2}\sigma_{w}^{4}NM[SNR^{2}+2(1+\beta^{2})]}\right]\\ \;-\frac{1}{\text{ρ}\sigma_{w}^{2}\sqrt{2\pi NM(1+\beta^{2})}}\exp\left[-\frac{THR_{GD}^{2}}{2{\text{ρ}}^{2}\sigma_{w}^{4}NM(1+\beta^{2})}\right]\;=\text{0 ; }\sigma_{\xi }^{\text{2}}\neq \sigma_{\eta }^{2}\; \end{array}\right.$$

At the condition SNR<<1 we can use the following approximations ${\left(SNR\right)}^{2}+4\approx 4$ and $SNR^{2}+2(1+\beta^{2})\approx 2(1+\beta^{2})$. Solving Equation (B6) with respect to the threshold $THR_{GD}$, the optimal GD threshold can be expressed as

(B7) $$\left\{ \begin{array}{l} \;THR_{GD}^{op}=\frac{4NM\sigma_{w}^{2}}{1+{\text{ρ}}^{-2}}\text{ ; }\sigma_{\xi }^{\text{2}}=\sigma_{\eta }^{2}=\sigma^{2}\\ \;THR_{GD}^{op}=\frac{4NM\sigma_{w}^{2}(1+\beta^{2})}{2(1+{\text{ρ}}^{-2})}\text{ ; }\sigma_{\xi }^{\text{2}}\neq \sigma_{\eta }^{2}\; \end{array}\right.$$

## References

[1] Sahai A., Hoven N., Tandra R. Some fundamental limits on cognitive radio. Proceedings of the 42nd Allerton Conference on Communication, Control, and Computing.

[2] Tandra R., Sahai A. Fundamental limits on detection in low SNR under noise uncertainty. Proceedings of the 2005 IEEE International Conference on Wireless Networks, Communications and Mobile Computing.

[3] Tandra R., Sahai A. (2008). SNR walls for signal detection. IEEE J. Sel. Top. Signal Process..

[4] Ji G., Zhu H. (2010). On the noise power uncertainty of the low SNR energy detection in cognitive radio. J. Comput. Inf. Syst..

[5] (2006). IEEE Std. 802.22–06/0088r0. Numerical Spectrum Sensing Requirements. http://www.ieee802org/22/Meeting_documents/2005_June/22-06-0088-00-0000_Numerical_Spectrum_Sensing_Requirements.doc.

[6] Mariani A., Giorgetti A., Chiani M. (2011). Effects of noise power estimation on energy detection for co-gnitive radio application. IEEE Trans. Commun..

[7] Sonnenschein A., Fishman P.M. (1992). Radiometric detection of spread spectrum signals in noise of uncertain power. IEEE Trans. Aerosp. Electron. Syst..

[8] Yu G., Long C., Xiang M., Xi W. (2012). A novel energy detection scheme based on dynamic threshold in cognitive radio systems. J. Comput. Inf. Syst..

[9] Jouini W. (2011). Energy detection limits under log-normal approximated noise uncertainty. IEEE Signal Process. Lett..

[10] Alik M.S.O., Kokkeler A.B.J., Klumpernik E.A.M., Smit G.J.M. (2011). Lowering the SNR wall for energy detection using cross-correlation. IEEE Trans. Veh. Technol..

[11] Tian T., Iwai H., Sasaoka H. (2012). Energy detection using pseudo BER based SNR estimation scheme in cognitive radio. Sci. Engendering Rev. Doshisha Univ..

[12] Guibene W., Turki M., Hayar A. Distribution discontinuities detection using algebraic technique for spectrum sensing cognitive radio networks. Proceedings of the 5th International Conference on Cognitive Radio Oriented Wireless Networks and Communications.

[13] Guibene W., Turki M., Zayen B., Hayar A. (2012). Spectrum sensing for cognitive radio exploiting spectrum sensing discontinuities detection. EURASIP J. Wirel. Commun. Network..

[14] Semiari O., Maham B., Yuen C. (2014). On the effect of I/Q imbalance on energy detection and a novel four-level hypothesis spectrum sensing. IEEE Trans. Veh. Technol..

[15] Deng R., Chen J., Yuen C., Cheng P., Sun Y. (2012). Energy-Efficient cooperative spectrum sensing by optimal scheduling in sensor-aided cognitive radio networks. IEEE Trans. Veh. Technol..

[16] Liu Y., Xie S., Yu R., Zhang Y., Yuen C. (2013). An efficient MAC protocol with selective grouping and cooperative sensing in cognitive radio networks. IEEE Trans. Veh. Technol..

[17] Tuzlukov V. (1998). A new approach to signal detection theory. Digital Signal Process..

[18] Tuzlukov V. (2001). Signal Detection Theory.

[19] Tuzlukov V. (2002). Signal Processing Noise.

[20] Shbat M.S., Tuzlukov V. (2014). Evaluation of detection performance under employment of the generalized detector in radar sensor systems. Radioengineering.

[21] Tuzlukov V., Eksim A. (2012). Generalized approach to signal processing in wireless communications: The main aspects and some examples. Wireless Communications and Networks: Recent Advances.

[22] Tuzlukov V. (2013). Communication Systems: New Research.

[23] Shbat M.S., Tuzlukov V. (2014). Definition of adaptive detection threshold under employment of the generalized detector in radar sensor systems. IET Signal Process..

[24] Shbat M.S., Tuzlukov V. (2013). Spectrum sensing under correlated antenna array using generalized detector in cognitive radio systems. Int. J. Antennas Propag..

[25] Kim S., Lee J., Wang H., Hong D. (2009). Sensing performance of energy detector with correlated multiple antennas. IEEE Signal. Process. Lett..

[26] Jun M., Li Y., Zhang R.A., Wang R. (2012). A new spectrum sensing algorithm based on antenna correlation for cognitive radio systems. Wirel. Personal Commun..

[27] Molisch A.F., Greenstein L.J., Shafi M. (2009). Propagation issues for cognitive radio. IEEE Proc..

[28] Sun H., Laurenson D., Wang C.X. (2010). Computationally tractable model of energy detection performance over slow fading channels. IEEE Commun. Lett..

[29] Chen X., Yuen C. (2013). Efficient resource allocation in rateless-coded MU-MIMO cognitive radio network with QoS provisioning and limited feedback. IEEE Trans. Veh. Technol..

[30] Loyka S.L. (2001). Channel capacity of MIMO architecture using the exponential correlation matrix. IEEE Commun. Lett..

[31] Maximov M. (1956). Joint correlation of fluctuative noise at outputs of frequency filters. Radio Eng..

[32] Chernyak Y. (1960). Joint correlation of noise voltage at outputs of amplifiers with nonoverlapping responses. Radio Phys. Electron..

[33] Cordeiro C., Challapali K., Birru D., Shankar S. (2006). IEEE 802.22: An introduction to the first wireless standard based on cognitive radios. J. Commun..

[34] Makarfi A.U., Hamdi K.A. (2013). Interference analysis of energy detection for spectrum sensing. IEEE Trans. Veh. Technol..

[35] Zhang W., Mallik R.K., Letaief K.B. (2009). Optimization of cooperative spectrum sensing with energy detection in cognitive radio networks. IEEE Trans. Wirel. Commun..

[36] Atapattu S., Tellambura C., Jiang H. Spectrum sensing via energy detector in low SNR. Proceedings of the IEEE International Conference on Communications (ICC 2011).

[37] Mishra S., Sahai A., Brodersen R. Cooperative sensing among cognitive radios. Proceedings of the 2006 IEEE International Conference on Communications (ICC’06).

[38] Mariani A., Giorgetti A., Chiani M. SNR wall for energy detection with noise power estimation. Proceedings of the 2011 IEEE International Conference on Communications (ICC’11).

[39] Digham F.F., Alouini M.-S., Simon M.K. On the energy detection of unknown signals over fad-ing channels. Proceedings of the 2003 IEEE International Conference on Communications (ICC’03).

[40] Ghasemi A., Sousa E.S. (2007). Spectrum sensing in cognitive radio networks: The cooperation-processing trade off. Wirel. Commun. Mob. Comput..

[41] Ghasemi A., Sousa E.S. (2007). Opportunistic spectrum access in fading channels through collaborative sensing. J. Commun..

[42] Gradshteyn D.C., Ryzhik I. (2007). Table of Integrals, Series, and Products.

[43] Alouini M.S., Abdi A., Kveh M. (2001). Sum of gamma variates and performance of wireless communications systems over nakagami-fading channels. IEEE Trans. Veh. Technol..

[44] Liang Y.C., Zeng Y., Peh E., Hoang A.T. (2008). Sensing-throughput tradeoff for cognitive radio networks. IEEE Trans. Wirel. Commun..

[45] Kate S.K., Navare S.D., Bandewar S.R. (2007). Engineering Mathematics-II.

[46] Papadopoulos A. (2005). Metric Spaces, Convexity and Non-Positive Curvature.

